# Diagnosis and phylogenetic analysis of bovine leukemia virus in dairy cattle in northeastern Brazil

**DOI:** 10.3389/fvets.2022.1080994

**Published:** 2023-01-13

**Authors:** José Gomes Pereira, Cândida de Assunção Silva, Lucas Diniz Silva, Cristian Alex Aquino Lima, Carla Janaina Rebouças Marques do Rosário, Ellainy Maria Conceição Silva, Maria do Socorro Costa Oliveira, Larissa Sarmento dos Santos Ribeiro, Hamilton Pereira Santos, Ana Lucia Abreu-Silva, Ferdinan Almeida Melo

**Affiliations:** Department of Pathology, State University of Maranhão, São Luís, Brazil

**Keywords:** EBL, bovine, PCR, viral diseases, genetic characterization

## Abstract

Enzootic bovine leukosis (EBL) is a chronic viral disease of wide distribution in cattle herds and may take several years for the first manifestation of clinical signs. Most animals do not present clinical signs. However, the economic losses are underestimated due to this disease. Thus, this work aimed to detect and characterize BLV in dairy cattle in the Maranhão state, northeastern Brazil. Blood samples were collected from 176 animals from 8 municipalities in the southeastern state of Maranhão. Bovine blood samples were subjected to DNA extraction and molecular diagnosis using nested PCR assays for BLV, targeting gp51 gene. Positive samples were then sequenced and then subjected to phylogenetic inferences. BLV DNA was detected in 16 cattle (16/176, 9.09%) in 4 municipalities. Phylogenetic analyzes showed that the sequence obtained clustered in a clade containing BLV sequences classified as genotype 6, with a high degree of support. Our data shows BLV occurrence in the Northeast of Brazil and the identification of genotype 6 in this region. These findings contribute to the molecular epidemiology of this agent in Brazil.

## 1. Introduction

Enzootic Bovine Leukosis (EBL) is a chronic viral disease of wide distribution in bovine herds, caused by the bovine leukosis virus (BLV) belonging to the Retroviridae family and Orthoretrovirinae subfamily (1, 2).

About 30–70% of infected animals may develop persistent lymphocytosis (PL), which observes an increase in circulating B lymphocytes. In addition, 0.1–10% of infected animals may develop lymphosarcomas, which has a fatal course (3, 4). Furthermore, once infected, animals remain carriers and are sources of lifelong shedding of the virus (5).

According to normative instruction 50 of September 24, 2013 of MAPA (*Ministry of Agricultur*e, Livestock and Supply/Ministry of Agriculture, Livestock and Supply) (6), EBL is one of the diseases that must be notified monthly. However, the disease remains unknown to most farmers, and a strict sanitary policy needs to be. These factors contribute to the wide dissemination in the Brazilian herd.

Clinical signs include digestive disturbances, inappetence, and weight loss. In addition, lymphosarcoma is reported in animals aged 4–8 years. It is most frequent in peripheral and internal lymph nodes but, it was also observed abomasum, heart, uterus, retrobulbar space, and epidural region of the central nervous system (3, 7–9).

Blood containing contaminated lymphocytes transferred from animal to animal by iatrogenic means is considered the main route of disease transmission; however, it is reported that vertical transmission of BVL can occur by the uterine route or even orally, through the ingestion of colostrum and milk-containing lymphocytes contaminated with BLV (10, 11).

The BLV genome is constituted by identical copies of single-stranded RNA, which is transcribed into a double-stranded DNA called a provirus. It encodes capsid structural genes (g*ag*), the RNA-dependent DNA polymerase (the reverse transcriptase) (*pol*) and viral envelope proteins (*env*) (12). For purposes of phylogenetic analysis, *env* genes are widely used. Furthermore, this gene encodes two surface glycoproteins, GP51 and GP30, which are essential for infectivity and immune evasion. In addition, these genes made it possible to perform BLV genotyping and characterize at most minuscule 10 virus genotypes worldwide (13–16).

Many serological studies carried out in Brazil demonstrate the exposure of animals to BLV in all regions of the country (17–23). Studies carried out mainly in the country's southeastern region evidenced the agent's circulation and the characterization of genotypes 1, 2, 5, and 6 (24–27).

In the state of Maranhão, only one serological survey showed exposure of dairy cattle to the virus, with a prevalence of 53.8% (28). Thus, this study aimed to investigate, using a molecular diagnostic technique the occurrence of BLV in cattle herds and to characterize the phylogenetic relationships of the sequence described for the first time in Maranhão, a state located in the Northeast of the country.

## 2. Materials and methods

### 2.1. Ethical considerations

The research project was approved by the Committee on Ethics and Animal Experimentation (CEEA) of the Veterinary Medicine Course at UEMA, Protocol No. 044/2017 CEEA/UEMA).

### 2.2. Study area

**Figure 1:**
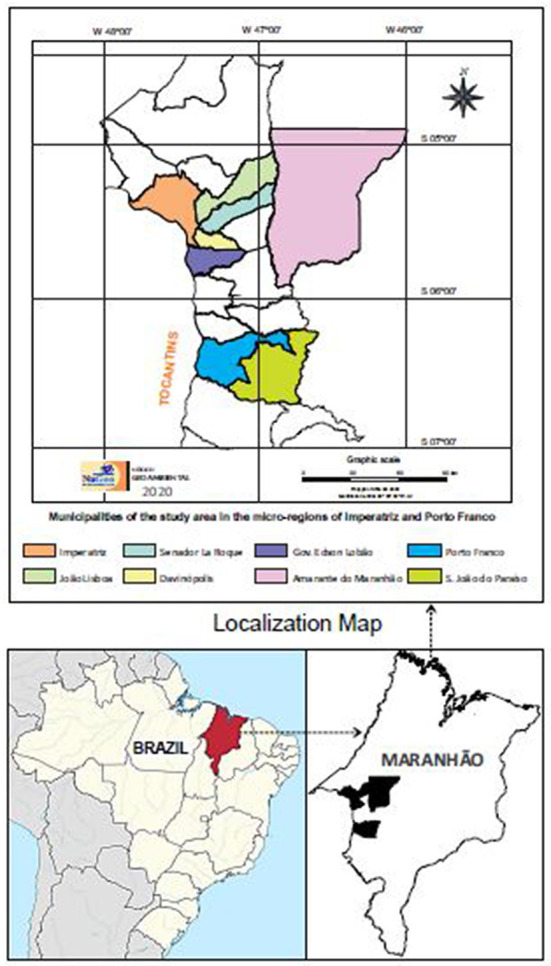
Map showing the location of municipalities where the sample collection was carried out. Locations are indicated by color.

The study was conducted in semi-intensive dairy cattle farms located in the rural zone of 8 municipalities in the southeastern region of the state of Maranhão: Imperatriz, Davinópolis, Porto Franco, Governador Edson Lobão, Amarante, Senador lá Roque, João Lisboa e São João do Paraíso (Figure 1).

The climate of this area is classified as hot and humid, with two well-defined seasons, rainy and dry. The average annual temperature in the region is about 26°C, with an annual amplitude of < 5°C, which characterizes the region's thermal regime with slight seasonal variation. This region is part of the cerrado biome (29).

### 2.3. Animals and sample collection

Blood collection was performed between August and September of 2018, during the dry season, from 176 randomly selected cattle. The animals' age ranged from 1 to 4 years, and dairy cattle, including females and calves, were all raised in a semi-intensive system.

Blood was obtained by venipuncture using vacutainer tubes with an anticoagulant. Then the samples were centrifuged at 1610 xG for 10 min to separate the buffy coat, which was removed and transferred to microtubes and stored at −20°C for other molecular biology techniques. No clinical sign was observed during the sample collection.

### 2.4. DNA extraction and conventional PCR for the endogenous *Cytochrome b* gene

For extraction of genomic DNA from the buffy coat, the commercial kit InstaGene™ Matrix (Bio-Rad Laboratories, Inc., USA), was used, following the manufacturer's instructions. To assess the quality, and the presence of extraction inhibitors and eliminate false negative results, all extracted DNA was subjected to PCR for the conserved *cyt b* of the mitochondrial DNA of vertebrates (mtDNA), according to the protocol described by Steuber et al. (30), which amplifies a 359 base pairs (bp) product.

The amplification reaction was performed using a final volume of 25 μL, using a mastermix solution containing 5.1 μL of Green GoTaq^®^ Flexi Buffer 5x (Promega, USA), 1.9 μL of each primer (5 pmol/μL–Sigma), 0.5 μL of dNTPmix (10 mM–Promega, USA) 1.5 μL of MgCl_2_ (10 mM–Promega, USA), 0.26 μL of Go TaqFlexi DNA Polymerase (500 U-Promega, USA), 2 μL of DNA template and DNase-free ultrapure water and RNase (Invitrogen-Life Technologies^®^, USA). The PCR cycles were programmed as follows: initial denaturation at 95°C for 5 min, 35 cycles composed of denaturation at 95°C for 15 s, annealing at 50°C for 30 s and extension at 72°C for 30 s, and final extension at 72°C for 5 min.

### 2.5. Nested-PCR for EBL based on the viral envelope *env (gp51)* gene

The samples positive in the *cyt b* gene were then subjected to n-PCR for detection of BLV, using primers described by Asfaw et al. (31), which amplifies a region of the *env* gene (gp51), of 444 bp. The first reaction had a final volume of 25 ul, with 3.0 μl of 10 mM of each primer, 0.4 mM DNTPs, 1X PCR buffer (Thermo Scientific^®^), 3 mM MgCl_2_ and 1U of Taq DNA polymerase. In the second PCR reaction, 5 μL DNA of the product of the first amplification was used as a template, with the same concentrations of the other reagents and the primers env 5099 (5' CCCACAAGGGCGGCGCCGGTTT-3') and env 5521 (5' GCGAGGCCGGGTCCAGAGCTGG-3'), in a final volume of 30 μL.

Both reactions (first and second) were carried out according to the following program: initial denaturation at 94 °C for 5 min, followed by 40 cycles of 94°C for 30 s, 60°C for 30 s and 72°C for 1 min, and final extension of at 72°C for 5 min. A BVL positive sample was used as a positive control and ultrapure water was used as a negative control in all reactions.

### 2.6. Electrophoresis

The amplified products were subjected to horizontal electrophoresis on a 1.5% agarose gel stained with ethidium bromide (0.5 μL/ml) in TAE buffer pH 8.3 (Tris Acetate EDTA). Electrophoresis was performed at 100 V and a current of 400 mA for 40 min. The results were visualized and analyzed by an ultraviolet light transilluminator coupled to a computer program for image analysis (L-PIX gels Loccus^®^).

### 2.7. Sample purification and sequencing

Amplified products positive for all genes tested were purified using the commercial Wizard^®^ SV Gel and PCR Clean-Up System (Promega^®^) kit, according to the manufacturers' recommendations. The quantification of purified DNA was performed using a NanoDrop 2000 spectrophotometer (Thermo Scientific, San Jose CA, USA). Sanger sequencing was performed according to the dideoxynucleotide chain termination method (32) on the ABI PRISM 3,500 Genetic Analyzer (Applied Biosystem, Foster City, CA, USA). The sequencing was performed at the ACTGene Molecular Analysis, Rio Grande do Sul, Brazil.

### 2.8. Phylogenetic analysis

The nucleotide sequence obtained was inserted into the FinchTV 1.4 software (Geospiza, Inc., Seattle, WA, USA) for evaluating the quality of electropherograms. In the Basic Local Alignment Search Tool program (33) the inserted sequence was compared with other sequences deposited in GenBank.

For phylogenetic analyses, gp51 sequences were aligned with other homologous sequences of the same sequenced gene, taken from the Genbank database, using the Clustal/W tool via Bioedit 7.2 (34). The best-fit model of sequence evolution was selected based on the Corrected Akaike Information Criterion (cAIC), using MEGAX (35). The phylogenetic trees were reconstructed based on the neighbor-joining (NJ) and maximum likelihood (ML) methods, with 1,000 replicates for bootstrap analysis. The sequences generated in this search were submitted to GenBank and assigned accession number MT135126.

## 3. Results

All samples amplified the endogenous *cyt b* gene, which was used for extraction evaluation. Thus, they were tested in nested PCR based on the *env* gene and in total 9.09% (16/176) of animals were positive. In total, 8 municipalities in the southeastern region of the state of Maranhão were included in this research, and of these, 4 had nPCR positive animals (Table 1).

**Table 1:** Percentage of animals positive for BLV by cities in the Southwest region of Maranhão.

**Cities**	**No. of samples**	**No. of positive animals**	**Percentage of animals positive for EBL**
Porto Franco	21	4	19.0%
Edson Lobão	20	0	0.0%
Amarante	20	4	20%
Senador La Roque	19	6	31.5%
João Lisboa	20	2	10%
São João do Paraíso	26	0	0.0%
Imperatriz	25	0	0.0%
Davinópolis	25	0	0.0%
Total	176	16	9.09

A sequence was obtained and used for phylogenetic analysis from positive samples. The genetic identity ranged from 96.85 to 100% with different BLV isolates from various locations in Brazil (JN254639, DQ059415, AY185360), South American countries such as Bolivia (LC075576), Paraguay (LC080656), and others countries like India (MH341525), Pakistan (MW926787), Russia (KC886631), Myanmar (LC466592), Thailand (KU233536).

**Figure 2:**
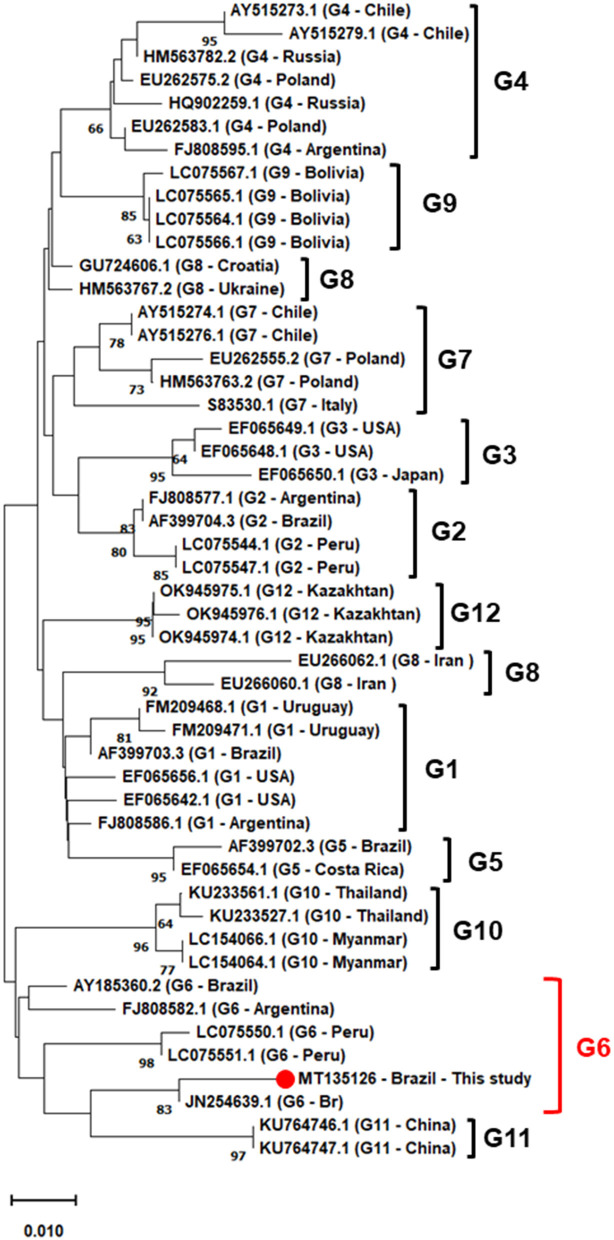
Phylogenetic inferences using the neighbor-joining (NJ) method based on the Kimura 2-parameter model, with gamma distribution (k2+G), the numbers at the branches show bootstrap support (1.000 replicates). The analysis involved 50 nucleotide *env* gene sequences from Brazil and the world, based on 464 bp fragment. The sequence described in this study is indicated by filled red circle. Evolutionary analyses were conducted in MEGAX.

Phylogenetic analysis was performed using ML and NJ methodologies, and both analyzes showed similar topology, so only the NJ is shown. For phylogenetic reconstruction, sequences of isolates from genotypes 1–12, deposited in the Genbank were selected. The analyzed sequence was grouped in a clade formed by BLV sequences belonging to genotype 6, with strong clade support. In this group, sequences from different countries were observed, such as Brazil, in São Paulo state, and other countries such Peru and Argentina all isolated from bovine blood (Figure 2).

## 4. Discussion

This study presents data on the occurrence and phylogenetic characterization of BLV in cattle herds in the southeastern state of Maranhão. This area has a critical livestock activity because it is the second largest cattle herd in the northeastern region of Brazil since it is a significant exporter of live cattle, ~1,000 heads/yearly (36).

According to the Ministry of Agriculture, Livestock, and Supply (*Ministério da Agricultura, Pecuária e Abastecimento*) (MAPA), enzootic bovine leukosis is mandatory monthly notification of confirmed cases (category 4). However, no specific public policies exist to control or eradicate the disease (6). Serological rates have varied between 11.78 and 45% in states in the northeast region (Pernambuco and Sergipe), in the midwest region (Mato Grosso do Sul) and in the southern region (Santa Catarina) (19–21, 37). In the state of Maranhão, there is only one survey showing the exposure of animals to BLV, and it showed high rates of 53.8% of antibodies (28).

On the other hand, surveys using molecular diagnostic techniques targeting the identification of viral DNA are even more limited. In southeastern and southern Brazil, respectively, D'Angelino et al. (26) reported 4.8% of CNS tissues from BLV-positive cattle in the state of São Paulo, and Rodakiewicz et al. (37), found a high rate of 80.5% (62/77) of positive animals in the State of Santa Catarina. Although serological studies are observed in the northeastern region, direct detection of virus DNA by molecular techniques has not been observed. This is the first molecular study, to our knowledge, conducted in the state of Maranhão.

Surveillance activities for the occurrence of BLV are very important, considering two aspects: the damage caused to infected animals and the zoonotic potential of BLV. The impacts caused by leukosis are expressive in the affected herds, as these animals maintain low production rates. Direct losses have been estimated due to reduced milk production, reproduction and premature slaughter of animals, and restrictions on international trade from countries where the virus is circulating (38–41). Because of this, it is crucial to have a current plan for the occurrence of BLV in Brazil, as it will allow a better understanding of the virus and the possible economic impacts in different regions.

It is important to mention that previous studies carried out in recent years have indicated the zoonotic potential of BLV. Although it has not yet been clearly determined how humans can be infected by the virus or its clinical implications, it has been associated with breast cancer in women (42, 43). Olaya-Galan et al. (44) reported the occurrence of BLV DNA in milk and meat products, demonstrating that human infection can occur orally. This fact reinforces the need to create BLV eradication programs in Brazil.

Regarding phylogenetic analyses, it was shown, using the NJ algorithm, that the sequence isolated from bovine in this research is clustered in a strongly supported clade, with isolates from bovines from São Paulo (JN254639) and Mato Grosso do Sul (AY185360) (100% bootstrap), in addition to sequences from Peru (LC05551 and LC05550) and Argentina (FJ808582). These results are similar to those already indicated in previous studies conducted in Brazil and Latin America, where genotype 6 seems to be very frequent. In addition to the South American continent, genotype 6 has been reported in Asian countries such as China (45, 46), Thailand (47), Myanmar (48), Vietnam (49), demonstrating the distribution of this genotype around the world. Sultanov et al. (50) point out that the introduction of infected cattle into herds is the main form of transmission of BLV, considering that the genotype 6 characterized in this locality is phylogenetically related to that found in the central-west and southeastern regions of Brazil, geographically distant, but that carry out the cattle trade with the state of Maranhão (36). This may have been an important route for the dissemination of genotype 6 in the country and also to other locations around the world. Brazil is one of the largest exporters of beef and lives cattle in the world; countries such as China, United States of America, Egypt, and Chile are the largest importers of Brazilian meat (51). Therefore, concentrated efforts to develop public policies to control enzootic bovine leukosis are essential to contain the spread of specific BLV genotypes.

## 5. Conclusions

BLV circulation was evidenced in dairy cattle in the state of Maranhão, Northeastern Brazil. For the first time, phylogenetic inferences showed genotype 6 in this region related to sequences previously identified in central-western and southeastern Brazil.

## Data availability statement

The datasets presented in this study can be found in online repositories. The names of the repository/repositories and accession number(s) can be found at: https://www.ncbi.nlm.nih.gov/genbank/, MT13512.

## Ethics statement

The research project was approved by the Committee on Ethics and Animal Experimentation (CEEA) of the Veterinary Medicine Course at UEMA, Protocol No. 044/2017 CEEA/UEMA). Written informed consent was obtained from the owners for the participation of their animals in this study.

## Author contributions

JP, FM, AA-S, MO, CR, LR, and HS: project idealization, experimental data interpretation, and writing. CS and LS: sample collection, hematological analyzes, data interpretation, and writing. CL and ES: helped molecular and experimental conduction. All authors read and approved the final manuscript.
